# Association between chronic sodium-glucose cotransporter 2 inhibitors use and right ventricular function in hemodynamically stable patients with acute pulmonary embolism

**DOI:** 10.3389/fcvm.2026.1975531

**Published:** 2026-10-26

**Authors:** Gassan Moady, Shir Maman, Robert Dragu, Lihi Levi-Gofman, Shaul Atar

**Affiliations:** 1 Department of Cardiology, Galilee Medical Center, Nahariya, Israel; 2 Azrieli Faculty of Medicine, Bar Ilan University, Safed, Israel

**Keywords:** acute pulmonary embolism, heart failure, outcome, right ventricle, sodium glucose cotransporter inhibitor

## Abstract

**Background:**

Acute pulmonary embolism (PE) is a common cardiovascular disorder associated with substantial morbidity and mortality, with prognosis largely determined by the extent of right ventricular (RV) involvement. Sodium-glucose cotransporter 2 (SGLT2) inhibitors improve outcomes in patients with heart failure (HF) and exert favorable effects on left ventricular (LV) remodeling; however, their effects on RV function in acute PE remain largely unexplored. We hypothesized that chronic treatment with SGLT2 inhibitors would be associated with a lower incidence of clinical congestion and a more favorable clinical course in patients with acute PE.

**Methods:**

We retrospectively compared 30 patients receiving chronic SGLT2 inhibitor therapy with 90 controls following 1:3 matching for multiple variables, including age, sex, comorbidities, and background medical therapy. Echocardiographic and clinical outcomes were compared between the groups, including tricuspid annular plane systolic excursion (TAPSE), systolic pulmonary artery pressure (SPAP), and clinical congestion.

**Results:**

Patients receiving SGLT2 inhibitors had higher TAPSE values (19.4 ± 2.7 vs. 17.3 ± 5, 95% Confidence interval (CI) 0.7–3.5, *p* = 0.004), fewer signs of clinical congestion [6.7% vs. 25.6%, Odds ratio (OR) 0.21, 95% CI 0.05–0.94, *p* = 0.03], lower troponin, [14.5 (5.3–51), vs. 64.5 (20–177), 95% CI (33–108), *p* < 0.001], and lower N-terminal pro-B-type natriuretic peptide (NT-proBNP) levels, [158 (96–443) vs. 1000 (333–3433), 95% CI (467–1153) pg/mL, *p* < 0.001]. No significant between-group difference was observed in 1-year readmissions due to dyspnea or clinical congestion [OR 0.34, 95% CI (0.1–1.2), *p* = 0.13].

**Conclusions:**

Among hemodynamically stable patients with acute PE, chronic SGLT2 inhibitor therapy was associated with more favorable RV function during the index event; however, this association did not translate into improved long-term clinical outcomes. These findings warrant confirmation in prospective studies.

## GRAPHICAL ABSTRACT

**Figure:**
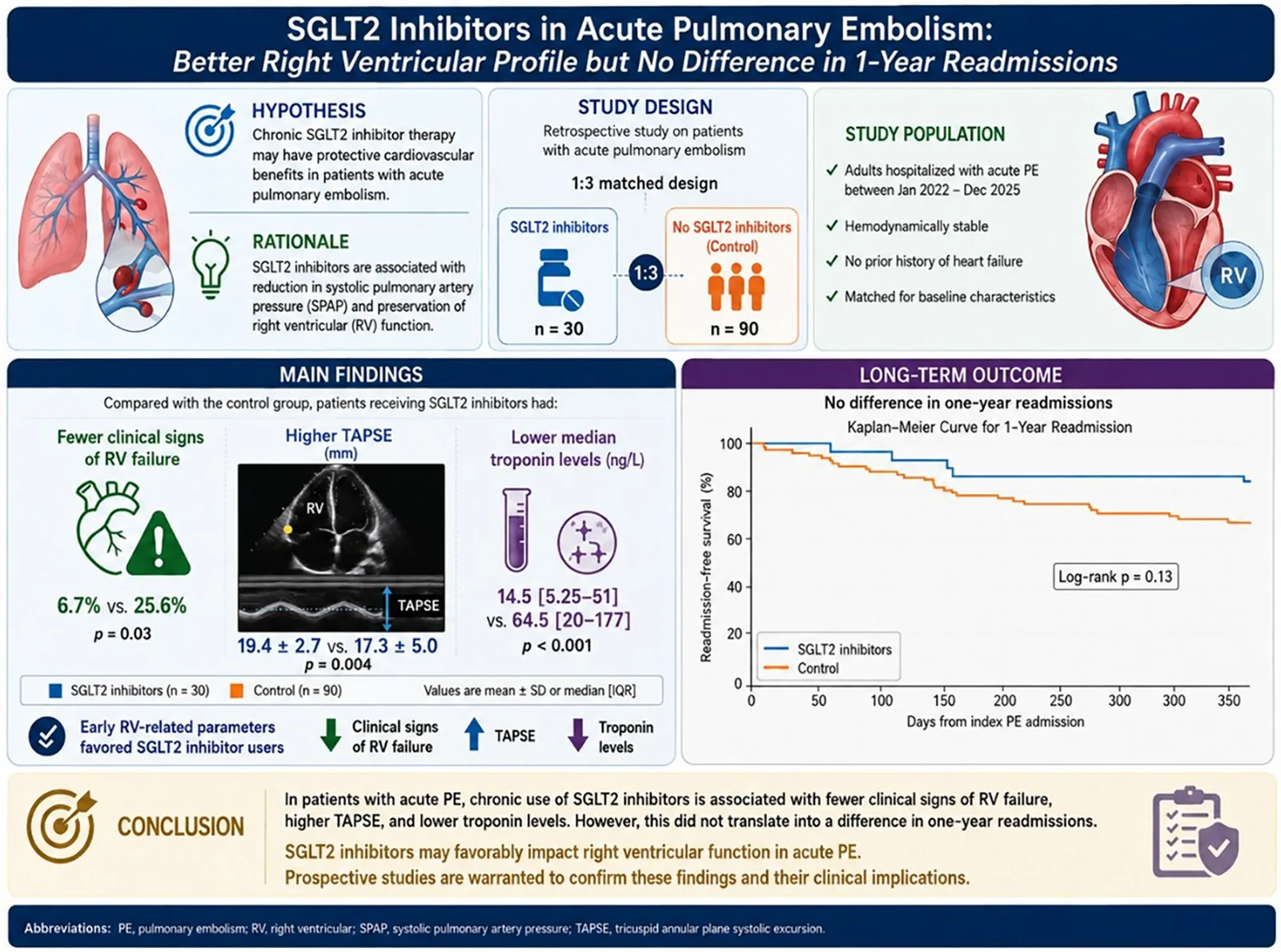
We assessed the association between chronic SGLT2 inhibitors on outcomes of patients with acute PE. We found that patients on SGLT2 inhibitors before the index hospitalizations had lower rate of clinical congestion, higher TAPSE, lower SPAP, and lower troponin levels, however, without effect on 1-year readmissions. We conclude that patients with acute PE treated with SGLT2 inhibitors may have favorable outcomes mediated by beneficial effects on RV function.

## 1 Introduction

Sodium glucose cotransporter 2 (SGLT2) inhibitors are considered the cornerstone therapy in patients with heart failure (HF) and chronic kidney disease (CKD) owing to favorable outcomes across the spectrum of cardiometabolic-kidney syndrome (1–4). This class of drugs exhibit their effects mainly through several pleiotropic pathways including improved cardiac metabolism, altered mitochondrial autophagy, enhanced diuresis, antifibrotic effects, and improved endothelial function (5–7). Previous studies have mainly targeted their effects on the left ventricle (LV) function and remodeling (8). Data on the impact of SGLT2 inhibitors on the right ventricle (RV) is currently limited to echocardiographic studies and clinical studies that were primarily conducted on chronic conditions such as chronic HF and adult congenital heart disease (9–11). Acute pulmonary embolism (PE) is a common cardiovascular disease that is associated with high morbidity and mortality rates (12). Risk stratification of patients with PE is mainly determined by hemodynamic condition, the degree of right ventricular (RV) involvement, and biomarkers of cardiac injury or wall stress e.g., troponin and N-Terminal Pro-B- Type Natriuretic Peptide (NT-proBNP) (13). RV function is typically evaluated during standard echocardiography using visual estimation of RV function, RV diameter and volume, and tricuspid annular plane systolic excursion (TAPSE) (14). In acute PE, sudden increase in systolic pulmonary artery pressure (SPAP) leads to RV dilatation and dysfunction which finally may dictate hemodynamic status (15). Currently, there are no studies that evaluated the safety and efficacy of SGLT2 inhibitors in conditions that affect the RV in the acute phase. Given their pleiotropic cardiovascular benefits, we hypothesized that SGLT2 inhibitors in the setting of acute PE would be associated with good safety profile along with favorable outcomes derived by preserving RV function and thereby preventing hemodynamic deterioration.

## 2 Methods

### 2.1 Study design and population

The study included patients hospitalized for acute PE between January 2022 and December 2025 in cardiology, intensive care, and intensive cardiac care units. The diagnosis of acute PE was confirmed by computed tomography angiography in most cases while perfusion imaging was requested in cases of severe iodine allergy or advanced renal failure. All patients were treated according to common practice in acute PE depending on hemodynamic status. Hemodynamic, laboratory and echocardiographic parameters were obtained during the first two days after admission. We included low risk and intermediate-low risk patients who were treated with oral anticoagulation. High risk patients and patients with intermediate-high risk with deterioration (and thereby treated with intravenous thrombolysis or endovascular ultrasound-facilitated interventions) were excluded. Patients with previous HF or LV dysfunction and patients with missing laboratory or echocardiographic data were also excluded. Between January 2022 and December 2025, we identified 632 patients with acute PE. We identified patients with chronic SGLT2 inhibitors before the index events. The minimum duration was two months with median time of four months. Simplified Pulmonary Embolism Severity Index (sPESI) was calculated based on six criteria (age>80, history of cancer, HF or chronic lung disease, heart rate>110, systolic blood pressure <100, and oxygen saturation <90%) with each worth 1 point. The study was approved by the local ethical committee. Number of approval: NHR-0044-25, date of approval: May/20/2025. The study flowchart is summarized in Figure 1.

**Figure 1:**
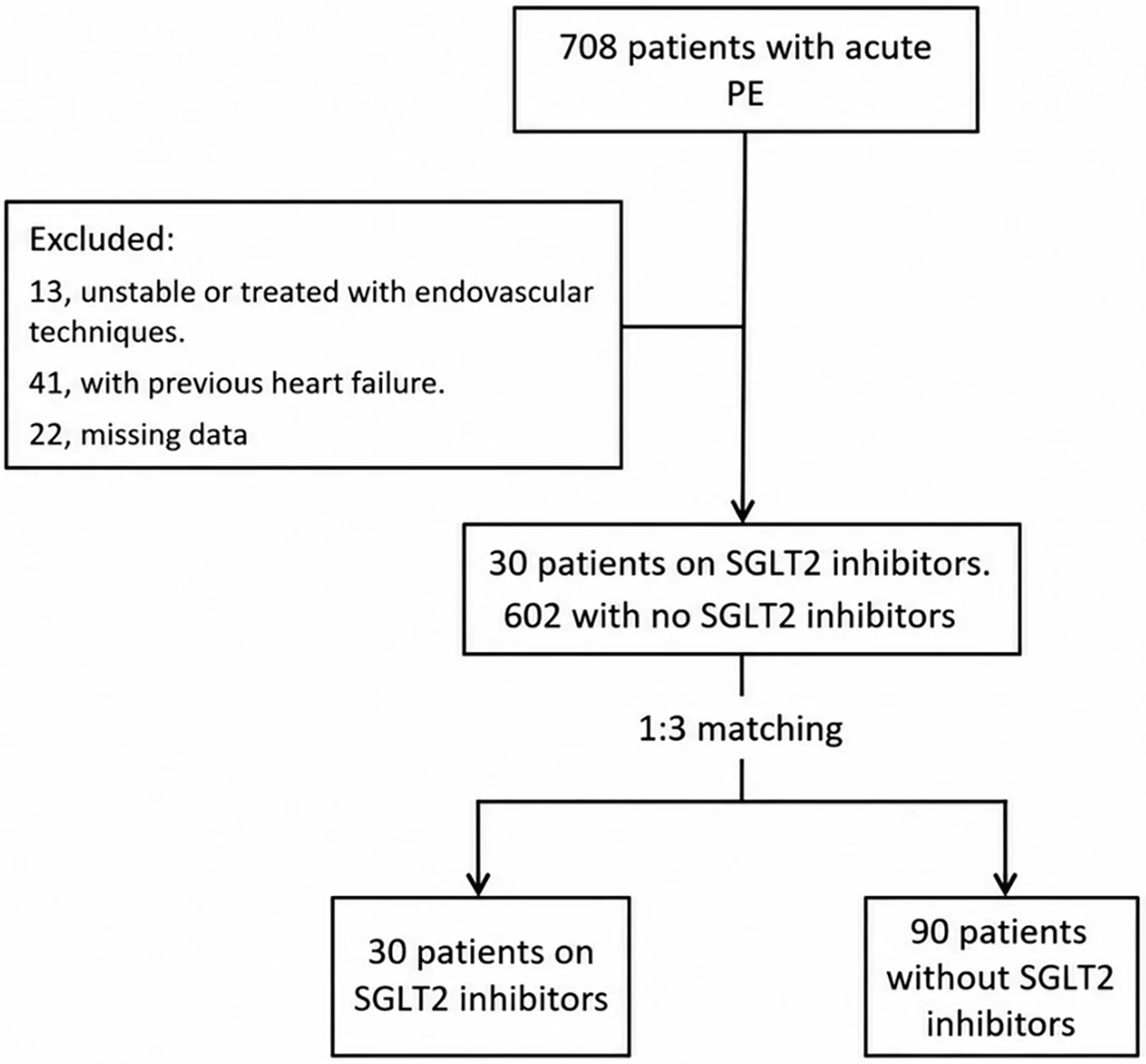
Study flowchart. Flowchart illustrating the study design. After excluding hemodynamically unstable patients, those with a history of heart failure, and those with missing data, 602 patients with acute pulmonary embolism were identified. Of these, 30 patients receiving SGLT2 inhibitors were matched to 90 controls in a 1:3 ratio. PE, pulmonary embolism; SGLT2, sodium- glucose cotransporter 2.

### 2.2 Echocardiography

All echocardiographic measurements were performed using Philips Epiq-7 machine and Epiq X8-2t transducer (Philips, Andover, MA, USA). Echocardiographic data was analyzed and calculated by expert cardiologist, specialized in echocardiography. Right atrial pressure (RAP) was estimated based on vena cave diameter and collapsibility. A normal-sized inferior vena cava (<2.0 cm) and normal respiratory variation with inspiration (>50% decrease) indicates normal right atrial pressure (<10 mm Hg). Mildly increased diameter (2.0–2.5 cm) or decreased respiratory variation signifies mildly elevated RAP (10–15 mm Hg), and a dilated inferior vena cava (>2.5 cm) with decreased respiratory variation signify highly-elevated RAP (>15 mm Hg). Continuous-wave doppler of the tricuspid regurgitation jet was carried out in the RV inflow, short axis, and apical four-chamber view. SPAP was calculated based on peak tricuspid valve regurgitation signal and estimated RAP using the formula SPAP = 4 (tricuspid valve regurgitation velocity)^2^ +RAP. TAPSE was determined in M-mode echocardiography by aligning the lateral tricuspid annulus with the ventricular apex in the apical four-chamber view and measuring the lateral annular displacement. RV dilatation was defined as basal diameter >41 mm in the apical four-chamber view. An illustrative summary for calculating SPAP is given in Figure 2.

**Figure 2:**
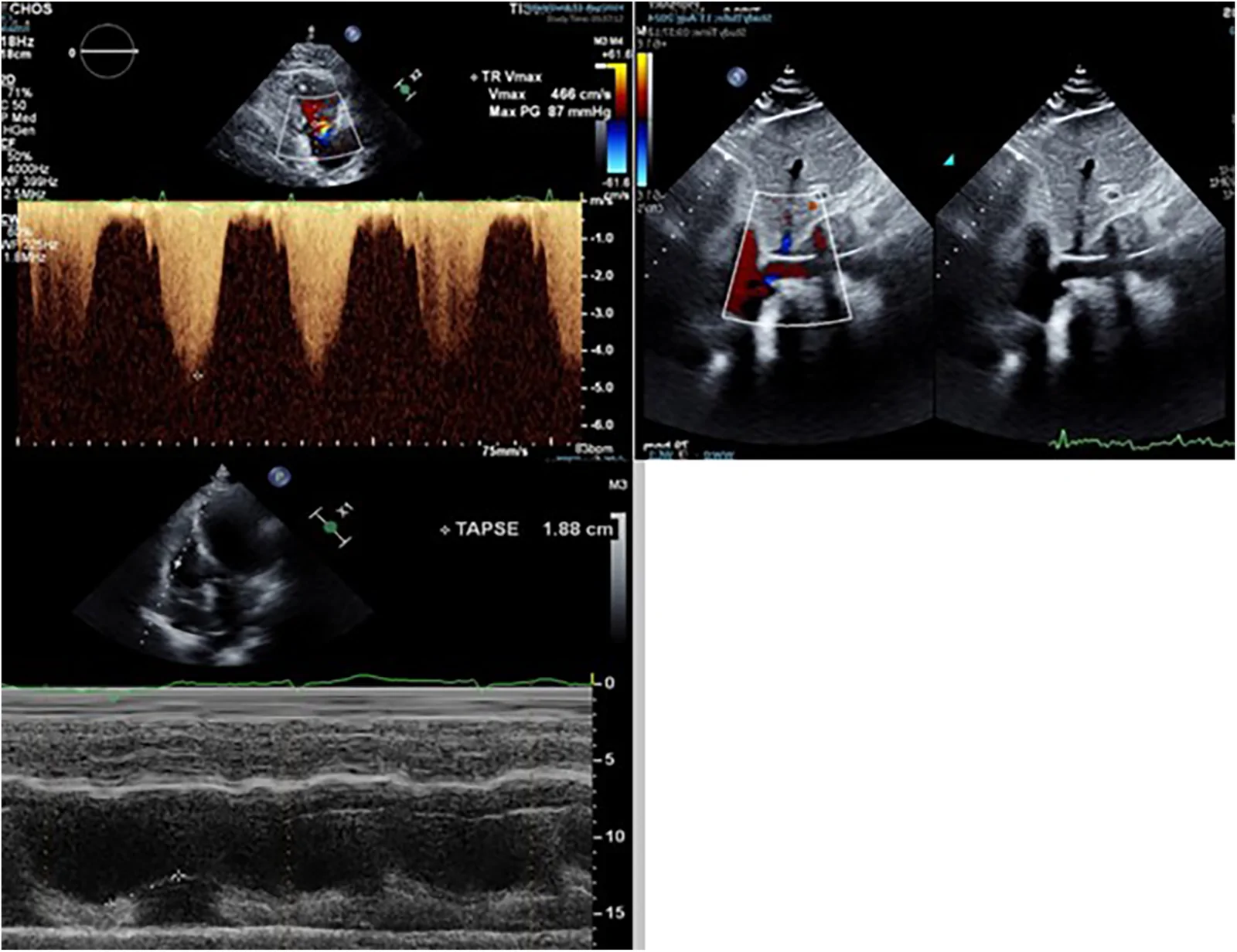
Illustration of SPAP and TAPSE calculation. Illustrative figure demonstrating the estimation of systolic pulmonary artery pressure (SPAP) based on tricuspid regurgitation peak velocity in the RV inflow view (upper left) and inferior vena cava diameter and inspiratory collapsibility in the subcostal view (upper right). Tricuspid annular plane systolic excursion (TAPSE) is measured in the apical four-chamber view using M-mode echocardiography (lower panel).

### 2.3 Outcomes

For each patient, we obtained data regarding clinical congestion including peripheral edema and jugular venous distension, the need for intravenous diuretic use. Echocardiographic outcomes included SPAP and TPASE, whereas hemodynamic parameters were systolic blood pressure and heart rate. One-year readmissions for dyspnea or clinical congestion were also documented and compared between the groups.

### 2.4 Statistical analysis

Data are presented as mean with standard of deviation (SD) or median with interquartile range (IQR) for continuous variables, and percentages for categorial variables. To reduce baseline differences between patients treated with SGLT2 inhibitors and controls, propensity score matching was performed. The propensity score was estimated using a logistic regression model in which treatment with an SGLT2 inhibitor was the dependent variable. The matching variables included age, sex, body mass index (BMI), background disorders, laboratory parameters, and baseline medical therapy. Complete nearest-neighbor matching was evaluated using 1:3 ratio. A caliper-based approach using 0.2 standard deviations of the logit of the propensity score was also explored as a sensitivity approach, but it retained only a limited number of treated patients and was therefore not used as the primary matched cohort. Covariate balance before and after matching was assessed using standardized mean differences. Balance was considered acceptable when the absolute standardized mean difference was less than 0.1, although variables with residual imbalance were also reported. Fisher's exact test and Welch's *t*-test or Mann–Whitney U test were used to compare categorial and continuous variables, respectively. Kaplan–Meier curve was used to illustrate readmissions in the two groups. All statistical tests were two-sided, and a *p*-value <0.05 was considered statistically significant.

## 3 Results

Among 632 with acute PE, thirty (4.7%) were on SGLT2 inhibitors before the index event. the baseline characteristics of the two groups before matching are given in Table 1.

**Table 1:** Baseline characteristics before propensity-score matching.

Variable	SGLT2 inhibitors group *N* = 30	Control group *N* = 602	Difference	*P* value
Age, years, mean ± SD	68.8 ± 11.1	67 ± 16	+1.8	0.404
Male, *n* (%)	13 (43.3)	284 (47.2)	−3.9 pp	0.712
BMI, kg/m^2^, mean ± SD	30.3 ± 4.8	31.4 ± 3.3	−1.1	0.224
Tobacco use, *n* (%)	5 (16.7)	150 (25)	−8.3 pp	0.388
Hyperlipidemia, *n* (%)	23 (76.7)	361 (60)	+16.7 pp	0.084
Diabetes mellitus, *n* (%)	26 (86.7)	207 (34.4)	+52.3 pp	<0.001
Hypertension, *n* (%)	23 (76.7)	418 (69.4)	+7.3 pp	0.541
Ischemic heart disease, *n* (%)	8 (26.7)	190 (31.6)	−4.9 pp	0.689
COPD, *n* (%)	3 (10)	37 (6.1)	+3.9 pp	0.428
CVA/TIA, *n* (%)	4 (13.3)	90 (15)	−1.7 pp	1.000
Chronic kidney disease, *n* (%)	7 (23.3)	186 (30.9)	−7.6 pp	0.425
Aspirin, *n* (%)	17 (56.7)	301 (50)	+6.7 pp	0.576
Statins, *n* (%)	21 (70)	373 (62)	+8.0 pp	0.443
RAAS inhibitors, *n* (%)	18 (60)	240 (39.9)	+20.1 pp	0.036
CCB, *n* (%)	5 (16.7)	84 (14)	+2.7 pp	0.597
Beta blockers, *n* (%)	15 (50)	271 (43.3)	+6.7 pp	0.708
Metformin, *n* (%)	23 (76.7)	335 (55.6)	+21.1 pp	0.024
Insulin, *n* (%)	9 (30)	150 (24.9)	+5.1 pp	0.522
GLP1-RA, *n* (%)	3 (10)	54 (9)	+1.0 pp	0.746
Diuretics, *n* (%)	4 (13.3)	108 (17.9)	−4.6 pp	0.631
Spironolactone, *n* (%)	2 (6.7)	48 (8)	−1.3 pp	1.000
Hemoglobin, g/dL, mean ± SD	12.7 ± 1.9	12.1 ± 2.3	+0.6	0.104
Creatinine, mg/dL, mean ± SD	0.83 ± 0.28	1.12 ± 0.6	−0.29	<0.001

Difference = SGLT2 inhibitor group minus control group; for categorical variables, differences are percentage points (pp). *P* values: Fisher's exact test for categorical variables and Welch's *t*-test for continuous variables.

BMI, body mass index; CCB, calcium channel blocker; COPD, chronic obstructive pulmonary disease; CVA, cerebrovascular accident; GLP1-RA, glucagon-like peptide-1 receptor agonist; RAAS, renin-angiotensin-aldosterone system; SD, standard deviation; TIA, transient ischemic attack.

The baseline characteristics of the two groups after 1:3 matching, are summarized in Table 2.

**Table 2:** Baseline characteristics of the two groups after propensity score matching.

Variable	SGLT2 inhibitors group *N* = 30	Control group *N* = 90	Difference	*P* value
Age, years, mean ± SD	68.8 ± 11.1	68.7 ± 13	0.008	0.93
Male, *n* (%)	13 (43.3)	41 (45.6)	0.045	1
BMI, kg/m^2^, mean ± SD	30.3 ± 4.8	29.4 ± 3.2	0.22	0.3
Tobacco use, *n* (%)	5 (16.7)	30 (33.3)	0.39	0.1
Hyperlipidemia, *n* (%)	23 (76.7)	61 (67.8)	0.19	0.49
Diabetes mellitus, *n* (%)	26 (86.7)	70 (77.8)	0.23	0.43
Hypertension, *n* (%)	23 (76.7)	66 (73.3)	0.08	0.81
Ischemic heart disease, *n* (%)	8 (26.7)	30 (33.3)	0.15	0.65
COPD, *n* (%)	3 (10)	5 (5.6)	0.17	0.41
CVA/TIA, *n* (%)	4 (13.3)	15 (16.7)	0.09	0.78
Chronic kidney disease, *n* (%)	7 (23.3)	31 (34.4)	0.25	0.37
Aspirin, *n* (%)	17 (56.7)	43 (48.3)	0.18	0.53
Statins, *n* (%)	21 (70)	46 (51.7)	0.39	0.092
RAAS inhibitors, *n* (%)	18 (60)	38 (42.2)	0.36	0.097
CCB, *n* (%)	5 (16.7)	10 (11.1)	0.16	0.52
Beta blockers, *n* (%)	15 (50)	39 (43.3)	0.13	0.53
Metformin, *n* (%)	23 (76.7)	61 (67.8)	0.19	0.49
Insulin, *n* (%)	9 (30)	12 (13.3)	0.41	0.052
GLP1-RA, *n* (%)	3 (10)	8 (8.9)	0.04	1
Diuretics, *n* (%)	4 (13.3)	20 (22.2)	0.23	0.43
Spironolactone, *n* (%)	2 (6.7)	6 (6.7)	0	1
Hemoglobin, g/dL, mean ± SD	12.7 ± 1.9	12.5 ± 1.8	0.11	0.58
Creatinine, mg/dL, mean ± SD	0.83 ± 0.28	0.93 ± 0.27	0.36	0.11

BMI, body mass index; CCB, calcium channel blocker; COPD, chronic obstructive pulmonary disease; CVA, cerebrovascular accident; GLP1 RA, glucagon-like peptide receptor agonist; RAAS, renin -angiotensin-aldosterone; SD, standard deviation; TIA, transient ischemic attack.

Patients in the SGLT2 inhibitors group have fewer clinical signs of clinical congestion (6.7% vs. 25.6%, *p* = 0.03), higher TAPSE [19.4 ± 2.7 vs. 17.3 ± 5, mean difference 2.1 mm, 95%CI (0.7–3.5), *p* = 0.004], lower median troponin [14.5, (5.3–51) vs. 65, (20–177); mean difference 50, 95% CI (33–108), *p* < 0.001] and NT-proBNP [158 (96–443) vs. 1,000 (333–3,433), mean difference 843 pg/mL, 95%CI (467–1153), *p* < 0.001]. No significant deference was observed in the percentage of patients with RV dilatation (13.3% vs. 27.8%, *p* = 0.14). It should be noted that sPESI scores were comparable between the groups [1 (0–2) vs. 1(0–2), *p* = 0.6]. The outcomes are summarized in Table 3.

**Table 3:** Echocardiographic parameters and clinical outcomes.

Variable	SGLT2 inhibitors group *N* = 30	Control group *N* = 90	*P* value
SPAP mmHg, mean ± SD	36.5 ± 7	39.2 ± 11.7	0.14
TAPSE cm, mean ± SD	19.4 ± 2.7	17.3 ± 5	0.004
Troponin ng/L, median [IQR]	14.5 [5.3–51]	64.5 [20–177]	<0.001
NT-proBNP pg/ml, median [IQR]	158 [96–443]	1000 [333–3433]	<0.001
SBP mmHg, mean ± SD	124.7 ± 17.0	129.2 ± 21	0.246
HR beats/min, mean ± SD	86 ± 15	91 ± 19	0.145
Clinical congestion, *n* (%)	2 (6.7)	23 (25.6)	0.03
RV dilatation, *n* (%)	4 (13.3)	25 (27.8)	0.14
IV diuretics, *n* (%)	5 (16.7)	20 (22.2)	0.6
DKA, *n* (%)	0	0	N/A

DKA, diabetic ketoacidosis; HR, heart rate; IQR, interquartile range; NT-proBNP, N-terminal pro-B-type natriuretic peptide; RV, right ventricular; SBP, systolic blood pressure; SD, standard deviation; SPAP, systolic pulmonary artery pressure; TAPSE, tricuspid annular plane systolic excursion.

There was no significant difference in one-year readmissions for dyspnea, chest pain, or recurrent thromboembolic events between the two groups [OR 0.34, 95% CI (0.1–1.2), *p* = 0.13]. Kaplan–Meier curve is depicted in Figure 3.

**Figure 3:**
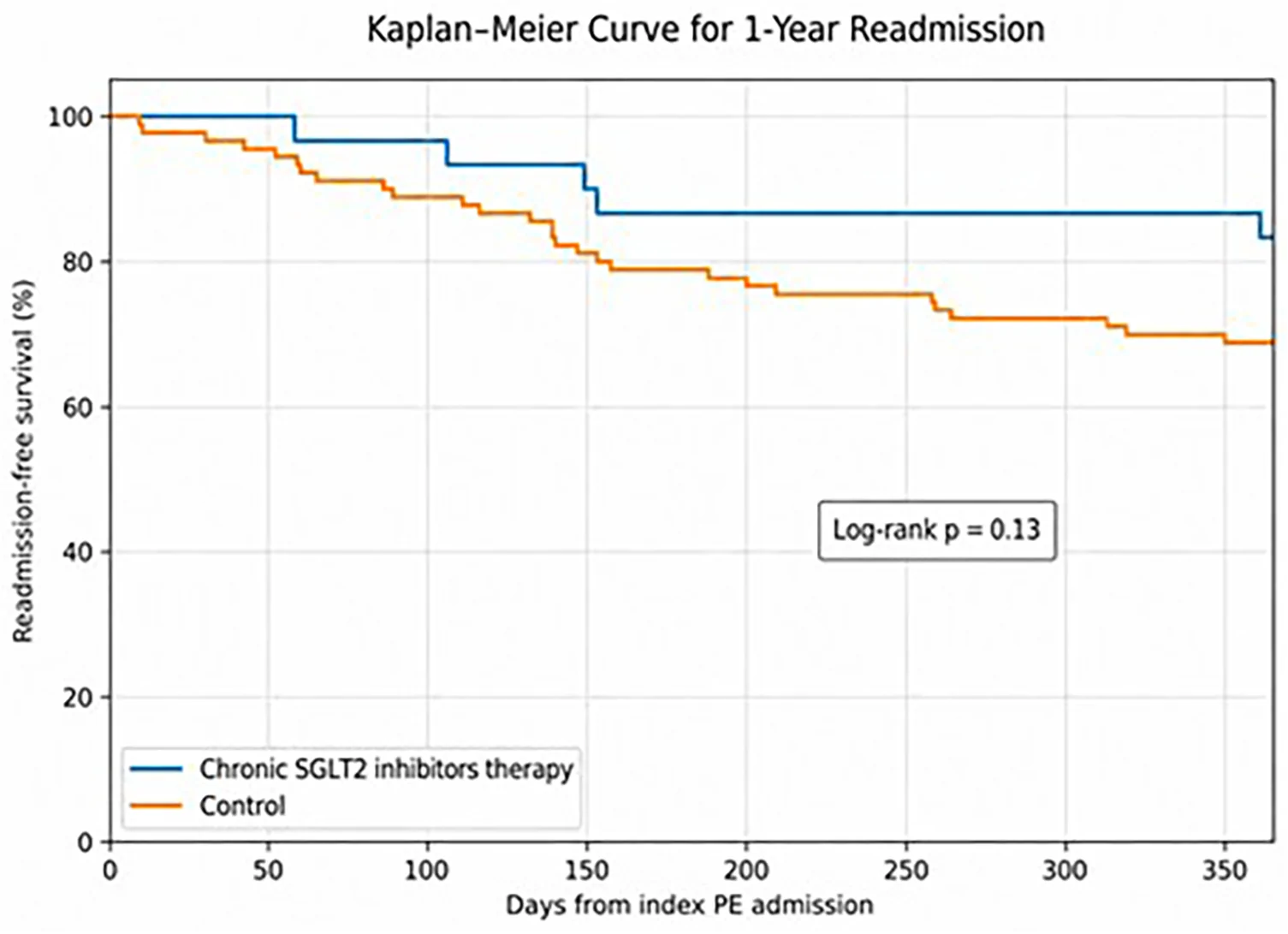
Kaplan–Meier curve for readmissions. Kaplan–Meier curve for 1-year readmissions of the two study groups following the index event. There was no significant difference in readmission rate (*p* = 0.13).

## 4 Discussion

In this retrospective, exploratory, hypothesis-generating study, we assessed the association between pre-existing SGLT2 inhibitor use and RV function and biomarker profile at presentation among patients with PE, based on the hypothesis that the cardiometabolic effects of SGLT2 inhibitors may help preserve RV function during the acute phase. Chronic SGLT2 inhibitor use was associated with a lower incidence of clinical congestion, higher TAPSE, and lower levels of cardiac biomarkers, however, this association did not translate into improved long-term clinical outcomes. To the best of our knowledge, this is the first study to investigate this issue. SGLT2 inhibitors generally reduce LV filling pressure, particularly in patients with HF and elevated baseline filling pressures. Their effects are mediated through several mechanisms, including reduced plasma and interstitial volume, lower LV preload, improved ventricular vascular interactions, and potentially improved myocardial relaxation and compliance (5). Therefore, a potential association between chronic SGLT2 inhibitor use and improved RV function in acute PE cannot be attributed solely to lower LV filling pressure. Other mechanisms, including altered volume status and cardiometabolic effects, may contribute, but direct protection against PE-induced RV dysfunction remains unproven. Our preliminary findings suggest a potential beneficial role of SGLT2 inhibitors in attenuating hemodynamic deterioration, possibly through preservation of RV function. Given the established favorable effects of SGLT2 inhibitors on LV function and remodeling (16, 17), we hypothesized that these agents may also improve RV performance in the acute setting of PE through multiple mechanisms beyond glucose lowering. SGLT2 inhibitors reduce preload and afterload by natriuresis, diuresis, and vasodilation, which may help lower pulmonary artery pressure and RV stress (16). In animal models of pulmonary hypertension, SGLT2 inhibitors have been shown to attenuate pulmonary vascular remodeling and RV hypertrophy and fibrosis (17). These effects may be mediated by anti-inflammatory mechanisms, including inhibition of nucleotide-binding domain-like receptor protein 3 (NLRP3) inflammasome activation, downregulation of several interleukin-mediated signaling pathways, modulation of mitochondrial autophagy, nutrient-sensing pathways, oxidative stress, and fibrosis (18). SGLT2 inhibitors may also inhibit pulmonary artery smooth muscle cell proliferation and migration through activation of adenosine monophosphate-activated protein kinase (AMPK), attenuation of glycolytic activity, and improvement in vascular compliance (19). Studies have also suggested that reductions in serum uric acid levels may contribute to the cardioprotective effects of SGLT2 inhibitors, although the relevance of this mechanism to RV dysfunction remains uncertain (20). Previous echocardiographic studies evaluating the effects of SGLT2 inhibitors on the RV have reported promising findings, including improvements in the RV myocardial performance index and pulmonary arterial stiffness, as well as reductions in SPAP (21–23). Kirschbaum et al. further demonstrated that the addition of SGLT2 inhibitor was associated with a sustained reduction in SPAP, assessed using continuous invasive hemodynamic monitoring in patients with HF and either reduced or preserved LVEF (24). However, these studies were conducted in chronic settings and primarily included patients with HF. The pleiotropic mechanisms through which SGLT2 inhibitors exert favorable cardiovascular effects are unlikely to be confined to LV function. In acute PE, the abrupt increase in pulmonary vascular resistance leads to RV dilatation and, through ventricular interdependence, a reduction in LV preload (25, 26), while SGLT2 inhibitors may help attenuate RV dilatation and SPAP elevation while improving RV perfusion. Although patients receiving SGLT2 inhibitors in the present study had higher TAPSE values and fewer signs of clinical congestion, SPAP and the rate of RV dilatation did not differ significantly between the groups. In acute PE, SPAP measurements may be influenced by pre-existing pulmonary pressures and by the timing of echocardiographic assessment relative to the index event. We also found no significant difference in diuretic use between the groups despite the higher incidence of clinical congestion in the control group. This may reflect the fact that the decision to initiate diuretic therapy is largely based on individual clinical judgment. Troponin and NT-proBNP are the two principal biomarkers used for risk stratification in acute PE, reflecting the extent of myocardial injury and ventricular wall stress, respectively. Alteration in the levels of these biomarkers is rapid, and reflets clinical course with persistent NT-proBNP elevation indicating ongoing RV dysfunction and worse outcomes (27). Additionally, time from baseline to peak levels of NT-proBNP has been shown to be associated with in-hospital adverse events among patients with intermediate-high risk PE (28). The combination of RV dysfunction, demonstrated by lower TAPSE, and elevated troponin identifies patients at higher intermediate risk who require closer monitoring. Readmissions after acute PE is common with almost half of patients being readmitted within the first one year (29). Readmissions are mainly related to recurrent venous thromboembolism, anticoagulation-related bleeding, or persistent dyspnea and chest pain secondary to incomplete thrombus resolution, persistent RV dysfunction, post-PE syndrome, or chronic thromboembolic pulmonary hypertension (29, 30). In the present study, although the findings suggest an overall beneficial effect on RV performance, no significant difference in readmission rates was observed between the groups. This may be attributable to the small sample size and the inclusion of only hemodynamically stable patients, who had relatively low overall readmission rates. Overall, SGLT2 inhibitors are considered safe and are associated with relatively few adverse effects across diverse patient populations, including those with HF, ischemic heart disease, and CKD. Studies have also suggested that SGLT2 inhibitors may be beneficial in patients with acute HF once hemodynamic stability has been achieved (31). Their early clinical benefits as demonstrated in previous pivotal trials support initiation during hospitalization soon after stabilization. These clinical benefits are derived mainly from pleiotropic effects (1–5). Diabetic ketoacidosis (DKA) is a relatively rare adverse event, particularly when SGLT2 inhibitors are avoided in patients at increased risk of this complication (32–34). Therefore, the absence of documented DKA episodes in the present study is not unexpected, given the established safety profile of SGLT2 inhibitors, the small sample size, and the hemodynamically stable study population. Because many patients with acute PE also have comorbidities such as diabetes and CKD, initiation of SGLT2 inhibitors after clinical stabilization may be reasonable in appropriately selected patients, considering their favorable safety profile and potential additional cardiorenal benefits. However, this approach requires further evaluation in prospective studies.

## 5 Limitations

Our study has several limitations that should be considered when interpreting the findings. First, an important limitation is the potential for residual confounding. Although propensity-score matching improved balance in measured baseline characteristics, the relatively small number of patients receiving chronic SGLT2 inhibitor therapy limited the number of covariates that could be incorporated into the model and may have reduced the ability of matching to fully account for differences between the groups. In addition, PE-specific variables reflecting acute disease severity were not included in the propensity model because several of these parameters represented study outcomes or were closely related to the acute RV response. Consequently, differences in unmeasured factors, including thrombotic burden, baseline RV function, and other determinants of PE severity, may have contributed to the observed associations. The findings should therefore be interpreted as hypothesis-generating rather than evidence of a causal protective effect of SGLT2 inhibitor therapy. Second, patients with hemodynamic instability were excluded from our study. However, this exclusion may have limited clinical implications, as SGLT2 inhibitors are generally withheld during critical illness. Therefore, our findings are most applicable to hemodynamically stable patients with acute PE, in whom continuation of chronic SGLT2 inhibitor therapy may be clinically relevant. Third, the analysis of readmissions assumed continued use of SGLT2 inhibitors after discharge and no crossover between the study groups; however, longitudinal treatment adherence and medication changes were not systematically verified. Fourth, Patients with a history of HF were excluded from the study; therefore, significant volume overload at baseline was considered unlikely. Although baseline NT-proBNP levels were unavailable, we hypothesize that they were comparable between the two groups. Nevertheless, the absence of baseline NT-proBNP measurements precludes confirmation of this assumption, and potential differences in baseline volume status cannot be entirely excluded. Fifth, our study lacks serial echocardiographic assessment following the index event. Future prospective studies should incorporate measurements of LV and RV function to assess recovery or permanent cardiac dysfunction. Despite these limitations, the proposed mechanistic rationale is biologically plausible, and the preliminary findings are hypothesis-generating. In hemodynamically stable patients with acute PE, continuation of SGLT2 inhibitor therapy appeared may be associated with preservation of RV function and a lower risk of clinical deterioration. The absence of documented DKA should be interpreted cautiously given the small sample size and the low-risk, stable study population. Overall, these findings support further prospective studies to evaluate the safety and potential clinical benefits of SGLT2 inhibitors in acute PE.

## 6 Conclusions

In this small retrospective study of patients with acute PE, we report an association between background SGLT2 inhibitor therapy and favorable effects on RV function and biomarkers, however, this association did not translate into improved long-term clinical outcomes. Future randomized controlled trials are warranted to confirm these findings.

## Data Availability

The raw data supporting the conclusions of this article will be made available by the authors, without undue reservation.
